# Assessing the performance of health technology assessment (HTA) agencies: developing a multi-country, multi-stakeholder, and multi-dimensional framework to explore mechanisms of impact

**DOI:** 10.1186/s12962-021-00290-8

**Published:** 2021-07-02

**Authors:** Robyn Millar, Alec Morton, Maria Vittoria Bufali, Sven Engels, Saudamini Vishwanath Dabak, Wanrudee Isaranuwatchai, Kalipso Chalkidou, Yot Teerawattananon

**Affiliations:** 1 Department of Management Science, University of Strathclyde Business School, Sir William Duncan Building (Level 7), 130 Rottenrow, Glasgow, G4 0GE UK; 2 Health Intervention and Technology Assessment Program, Ministry of Public Health, Nonthaburi, Thailand; 3 Institute of Health Policy, Management and Evaluation, University of Toronto, Toronto, Canada; 4 Global Health at Imperial College London, London, UK; 5 Director of Global Health Policy and Senior Fellow at the Centre of Global Development, London, UK; 6 Saw Swee Hock School of Public Health, National University of Singapore, Singapore, Singapore

**Keywords:** Health technology assessment, Health technology assessment agency, Evaluation

## Abstract

**Background:**

Health technology assessment (HTA) agencies have an important role to play in managing the rising demands on health systems. However, creating and running such agencies potentially diverts resources from frontline services. A large number of studies address the question of ‘what is the impact of HTA?’. Several points of heterogeneity in this literature include: purpose of the study, definition of HTA, definition of impact, and scope and rigour of evaluations. Our study seeks to address several limitations in this literature. This study aims to explore the mechanisms of impact of an HTA agency. In doing so, we consider HTA as an institution rather than a knowledge product to build an impact evaluation framework from an international, multi-stakeholder and multi-dimensional perspective.

**Methods:**

We conducted 9 key informant interviews with experts from the international HTA community. We addressed several questions, informed by existing frameworks of impact within the literature, to understand their perspectives on the mechanisms of impact of an HTA agency. We analyse data using logic modelling and impact mapping, as tools to understand and visualise mechanisms of change.

**Findings:**

Our impact mapping highlights several distinct, but not necessarily mutually exclusive, mechanisms through which the overall impact of an HTA agency is achieved. These are: the effective conduct of HTA studies; effective use of HTA in agenda-setting and policy formulation processes; effective engagement and external communications; good institutional reputation and fit within the healthcare and policy-making system; effective use of HTA as a tool for the negotiation of health technology prices; and the effective implementation of policy change regarding health technologies. We also identify indicators of these effects.

**Conclusions:**

Our findings and resulting evaluation framework complement and add to existing literature by offering a new perspective on the mechanisms by which HTA agencies generate impact. This new perspective considers HTA as an institution rather than a knowledge product, is international, multi-dimensional, and includes multi-stakeholder views. We hope the analysis will be useful to countries interested in managing HTA performance.

**Supplementary Information:**

The online version contains supplementary material available at 10.1186/s12962-021-00290-8.

## Background

Health technology assessment (HTA) is a field of multidisciplinary research that aims to inform policy decisions and clinical practice around the use, introduction and reimbursement of health technologies. It uses specific methods to examine the health and social value, as well as cost implications of and ethical issues related to the use of a health technology in a systemic, transparent, unbiased and robust manner to inform decision-making [1, 2]. The overall goal of HTA research is to promote an ‘equitable, efficient, and high-quality health system’ [2]. HTA research can be conducted both by private (e.g. ICER in the United States) and by public actors, and the evidence HTA provides can inform decision makers about how best to ensure the health system is equitable, efficient, and of high quality.

While HTA research conducted by private actors can be informative to policy makers, several countries prefer to rely primarily on advice from publicly-funded national ‘HTA agencies’ that aim to serve the public interest. Two examples of such agencies are the HTA agencies of England (the National Institute for Health and Care Excellence, or NICE) and Thailand (the Health Intervention and Technology Assessment Program, or HITAP). Although the authority and responsibility given to HTA agencies varies from country to country, their prevalence across widely differing health systems is indicative of the power of HTA to add value across different contexts.

The launch of the UN’s Sustainable Development Goals (SDGs) in 2015 and its target to ‘achieve universal health coverage *(UHC)* […] for all’ [3] has drawn further attention towards the establishment of national HTA agencies, as they can offer a pathway to achieving and sustaining UHC even in severely resource-constrained environments. In particular, HTA’s rationale of directing resources towards health technologies that are ‘cost effective’—i.e. those that lead to large improvements in population health relative to the cost involved—allows nascent UHC initiatives to rapidly improve population health even under tight budgets.

However, with cost-effectiveness being such a crucial pillar of HTA, questions on the value added from investments into HTA agency capacity are inherently valid. After all, public resources invested in HTA agencies could have been diverted to frontline medical services offering much more ‘tangible’ health outcomes. Addressing the question of ‘value offered’ by HTA agencies is however not straightforward for a number of reasons. One reason for this is that existing HTA agencies around the world are highly heterogeneous, each operating in a different context, within different systems and under different budgets. As a result, the emerging impacts and externalities of HTA agencies may vary from context to context, highlighting the need to understand the question of *how* such agencies have impact.

To further position our study, we first discuss some key points from the literature to highlight the heterogeneity relating to the question “what is the impact of health technology assessment?”. The key points discussed put into context existing practice in the evaluation of impacts of HTA. We highlight a number of gaps in existing methodologies and approaches to the evaluation of HTA impact.

### The heterogeneous nature of evaluating the impact of health technology assessment

There are a large number of studies relating to the question “What is the impact of health technology assessment?”. However, since this question is rather poorly defined, the literature is quite heterogeneous. To review in detail would require more space than we have here and, in any case, others have been here before us [4–7]. Specifically, a good starting point for accessing this literature is the study by Gerhardus et al. [6] which provides a helpful framework and clear summary of the literature up to about 2006. Another useful resource is Raftery et al. [4] which gives a detailed description of the various methodologies which have been deployed to measure the impact of health research with a view to evaluating the impact of HTA. Rather than summarising these papers immediately, we will highlight their main contributions to further position our study.

In what follows, we highlight some points for discussion around the heterogeneity of this body of literature. Specifically, we highlight four sources of heterogeneity: (1) variations in the purpose of the study; (2) differences in interpretation of “health technology assessment”; (3) differences in interpretation of “impact”; and (4) variability in scope and level of rigour of evaluation studies. We conclude by highlighting what we think we can and cannot learn from this literature to position our study.

### Purpose of study

One source of heterogeneity in the literature is the purpose of the study. Some studies are relatively modest in aim, and are essentially descriptive, concerned with providing basic information about a sample of HTA reports and their findings [8–13]. Others have a stronger summative and analytical focus on the question of whether the investment in a health technology was worthwhile, in that they use either quantitative or qualitative data to explore the context in which the value is realised in more detail [14–16]. Yet other studies have a more formative purpose: how can the HTA system do a better job of delivering impact [17–19]? The nature of the evaluation assessment team also varies. Studies by external assessors, such as those commissioned to independent committees or pool of experts to evaluate the impact of HTA are often summative in nature, whereas studies by HTA or health system insiders are often descriptive or formative.

### Definition of the term “health technology assessment”

Further heterogeneity stems from ambiguity in the use of the term “health technology assessment”. Some researchers frame the question as being one of the impact of “reports” (or “guidance” or “advice”) from an HTA agency [6, 8–13, 16, 18–21]. Others frame the question as being one of the impact of HTA “research” [4, 14, 18, 22–26]. These are not necessarily the same. “Reports” may be based on an overview of a relatively small body of evidence generated elsewhere, whereas “research” implies a piece of work which is of publishable standard. Research itself may not necessarily lead to a report (for example if its main conclusion is that the status quo should be maintained). The tendency to frame the impact of HTA as being about the impact of some sort of knowledge product (whether report or research) is helpful for tracing impacts (as the knowledge product provides a source to which impact can be tracked) but arguably means that the more diffuse benefits of a visible HTA presence (e.g. encouraging evidence-based practice; legitimising discussions about cost-effectiveness) are relatively neglected.

### Definition of impact

Studies also differ in their interpretation of “impact” and conceptualisation of how impact occurs. For example, Gerhardus et al. [6] offer a six-stage model of impact which we paraphrase here:

1. Awareness: the relevant stakeholder must know of the HTA report.
2. Acceptance: the relevant stakeholder must see the HTA report as valid and a legitimate basis for action.
3. Policy process: the policy process should explicitly utilise the HTA report.
4. Policy decision: the policy decision should cite the HTA report.
5. Practice: there should be “clear and measurable” changes in clinical practice in line with policy decision and thus the report.
6. Outcome: health and economic outcomes should be realised on the basis of the changes in practice.

This six-stage model suggests that an ideal evaluation of the impact of HTA would provide evidence at all stages, and thus show that there was a clear chain from HTA study to health and economic outcomes. However, most studies omit some stages of this chain; some leave off the latter stages and some skip stages altogether. For example, studies which we designate as “model-based” studies [12, 16, 23] effectively skip the implementation chain almost entirely and provide estimates of health and financial benefits based on the HTA agency’s own cost-effectiveness studies, whether or not they have led to policy changes. This predominant focus on the endpoint of the chain characterises other types of evaluations, which appraise outcomes after changes in policy and practice occurred, rather than assuming that they will take place. This also applies to post-market field evaluations [20], where health technologies are assessed under real-world circumstances, or to studies that retrospectively analysed the longitudinal correlation between investments in health research and disease burden [24, 25]. Other studies integrate changes in policy and practice within the assessment, using varied approaches. For instance, primary or secondary data have been used to ascertain the extent to which preliminary HTA findings are actually implemented, thus adjusting pre-implementation model-based estimates to account for actual uptake and coverage [27, 28]. In other cases [8, 9, 11, 13, 19], analyses have assessed to what extent actual clinical practice and usage patterns adhered to guidance issued or appropriate use criteria. Others [29] primarily focussed instead on the time lag between HTA appraisal processes, policy decisions and, access for patients to approved medicines.

### Scope and rigour of evaluations

Studies also differ in terms of their scope and level of rigour. There is a trade-off between scope and level of rigour: the most rigorous or in-depth analyses are often those which focus exclusively on a single or narrow set of HTA recommendations, and are published in clinical journals for a particular medical sub-speciality [30, 31]. By single or narrow set of HTA recommendations, we mean that studies often have, without clear explanation, focused on the impact of specific recommendations, rather than the impact of the HTA agency as a whole. At the other end of the scale, studies which focus on the impact of a broader set of HTA reports [11, 12, 28, 32, 33] are inevitably somewhat broad-brush.

From our perspective an effective study design would use a mix of methods, with a quantitative component using state-of-the-art-statistical techniques to detect changes in system behaviour [13, 19, 31], and a qualitative component which draws on knowledge from a wide range of system stakeholders [10, 16]. Such a study design would also have a plausible answer to the question of how changes in system behaviour are attributed to the HTA agency (for example by comparing between territories where the agency’s jurisdiction does and does not hold), and would fully account for all residual uncertainties and list all background assumptions [12, 13, 16, 26, 34].

In terms of study scope, it is also worth noting that the most of studies addressing the value of HTA are context-specific, in that they generally focus on a unique HTA agency or national system. Conversely, in a few cases, studies adopt a comparative perspective. These either contrast the performance of multiple HTA bodies operating within a country [29], or generate insights by applying a common evaluative framework across multiple national settings, though usually narrowing down the number of HTA recommendations considered [27, 30].

### Summary of key points from the literature

We know, from the studies discussed in the previous section, that HTA studies have been conducted in several countries, and in many cases have influenced clinical practice and that there is also reasonably plausible evidence that—especially in medium-to large-sized countries—the benefits from implementing individual HTA recommendations can exceed the costs of performing the individual HTA by a substantial margin [15, 16, 20, 26–28, 35]. Moreover, the literature offers a rich resource of practical examples on how countries can evaluate their own HTA systems. However, we do not know everything. As highlighted above, from the standpoint of methodological perfection even the most rigorous studies have failings; there are significant gaps in terms of the coverage of time and space in the literature; and, as Raftery et al. [18] highlight, the available empirical literature is unlikely to be a random sampling of the entire human experience of HTA and most likely focusses on settings where HTA has been relatively successful.

### Study aim

In this study, in the interests of moving forward, we will focus on addressing two main limitations in the knowledge base. Firstly, current studies are typically at the country level and there is limited ability to cross compare between countries due to variations in reporting. However cumulative knowledge building would be greatly advanced if there were at least a few minimally accepted indicators for evaluating the impact of an HTA agency which could also be used for international comparisons. Secondly, available frameworks for the evaluation of HTA reports (such as the Gerhardus et al. [6] framework above) tend to be somewhat “linear” (in the words of Raftery et al. [4]) and focussed on HTA as a ‘knowledge product’ leading inexorably to change in health service practice and thus health and economic outcomes. However, this is not really compatible with what has been observed in several contexts with an HTA agency operating at the centre of a system and interacting with various stakeholders, as presented in the introduction. As a result, in this study we aim to explore the pathways to, or mechanisms which lead to the impact of an HTA agency from an institutional perspective. This perspective allows us to explore rules of behaviour (both formal and informal) that influence the impact of HTA agencies [36]. The aim of doing so is to build an HTA agency impact evaluation framework that complements existing research in this space. Furthermore, such an approach allows us to take an in-depth analysis not only of an HTA agency’s structure but also of its broader institutional surroundings and how these contribute to its value added. We believe that this study will be useful not only for assessments of organisation-level HTA impact, but that it can also help guide the design and developments of HTA systems globally.

## Methods

We conducted a qualitative study using key informant semi-structured interviews to capture the perspectives of 9 senior figures in the international HTA community. We focused on capturing their perspectives on the mechanisms of impact of having a national HTA agency. Specifically, interviewees have backgrounds and experience in several different national contexts that include Australia, Canada, Thailand, and United Kingdom. As a result of the international focus, participants were involved with different types of HTA agencies including those with a distinct decision-making capacity (e.g. in Australia). On the other hand, others participants reflect HTA agencies where HTA report and guideline development are the primary focus. Many of the participants, whilst being based within a specific HTA agency, had international HTA agency experience and as such were able to reflect more critically on the impact of HTA agencies at a more international and strategic level. Moreover, participants reflected a variety of perspectives within the HTA community including: academics, HTA specialists, and those with a clinical background.

### Ethics

The relevant ethical approval was granted from the ethics committee of the lead author’s institute. In keeping with the relevant ethical guidelines, participants received a participant information sheet informing them of the purpose and requirements of the study. At the beginning of the interview, the interviewer asked for verbal consent for each interview to be audio recorded and each interviewee’s identifying details were anonymised.

### Participant recruitment

Participant recruitment used a purposive sampling approach in order to select respondents based on their ability to provide the needed information [37]. We recruited respondents through contacts within the project team, and subsequently used a snowball sampling technique, which helped us to recruit respondents who otherwise would not have been accessible [38]. The national contexts within which interviewees had specific experience were predominantly contexts in which HTA agencies are well developed or had been in place for a relatively long period of time. We anticipated that respondents from these countries would have a rich understanding of the impact of HTA agencies, key stakeholders, and the factors, or mechanisms, influencing the overall impact of HTA agencies over an extended period of time.

### Development of an interview guide

We developed a semi-structured interview guide informed by our learning from several of the frameworks we discussed in the background section. We primarily informed our approach using the model provided by Gerhardus et al. [6] which outlines a six-stage model of impact. We also use insights from the Payback Framework, a popular framework discussing the impact of health services research [39]. The resulting interview guide focussed on asking about interviewee experiences of impact in terms of several effects of HTA from an institutional perspective. We list these below:

i. The use and effects of HTA studies (in terms of knowledge development and future research) in HTA agencies;
ii. The effects on policy and decision-making processes of having an HTA agency;
iii. The effects *from those* policy and decision-making processes of having an HTA agency on policy;
iv. The effects on the health sector of having an HTA agency;
v. The effects on health outcomes;
vi. The wider economic effects of having an HTA agency.

We used the responses to these questions to probe how and why participants thought that effect(s) had occurred, focussing on the factors that they thought influenced the realisation of those effect(s).

### Data collection and analysis

Interviews were conducted by a project team member via Skype and telephone call and were audio recorded subject to verbal participant consent. The data collection phase was followed by a two-stage data analysis phase using logic modelling and impact mapping.

### Logic modelling

Logic models were chosen as the tool for data analysis due to their ability to both visualise pathways to change and their use within the programme evaluation field. Logic modelling is based on the understanding of how programme activities contribute to changes in outcomes and overall impact for programme stakeholders [40]. Logic models are word-and-arrow diagrams that reflect the inputs, activities, outputs, and outcomes of a change initiative and their format is flexible so as to allow for the multi-dimensional and multi-stakeholder perspective adopted.

**Fig. 1:**
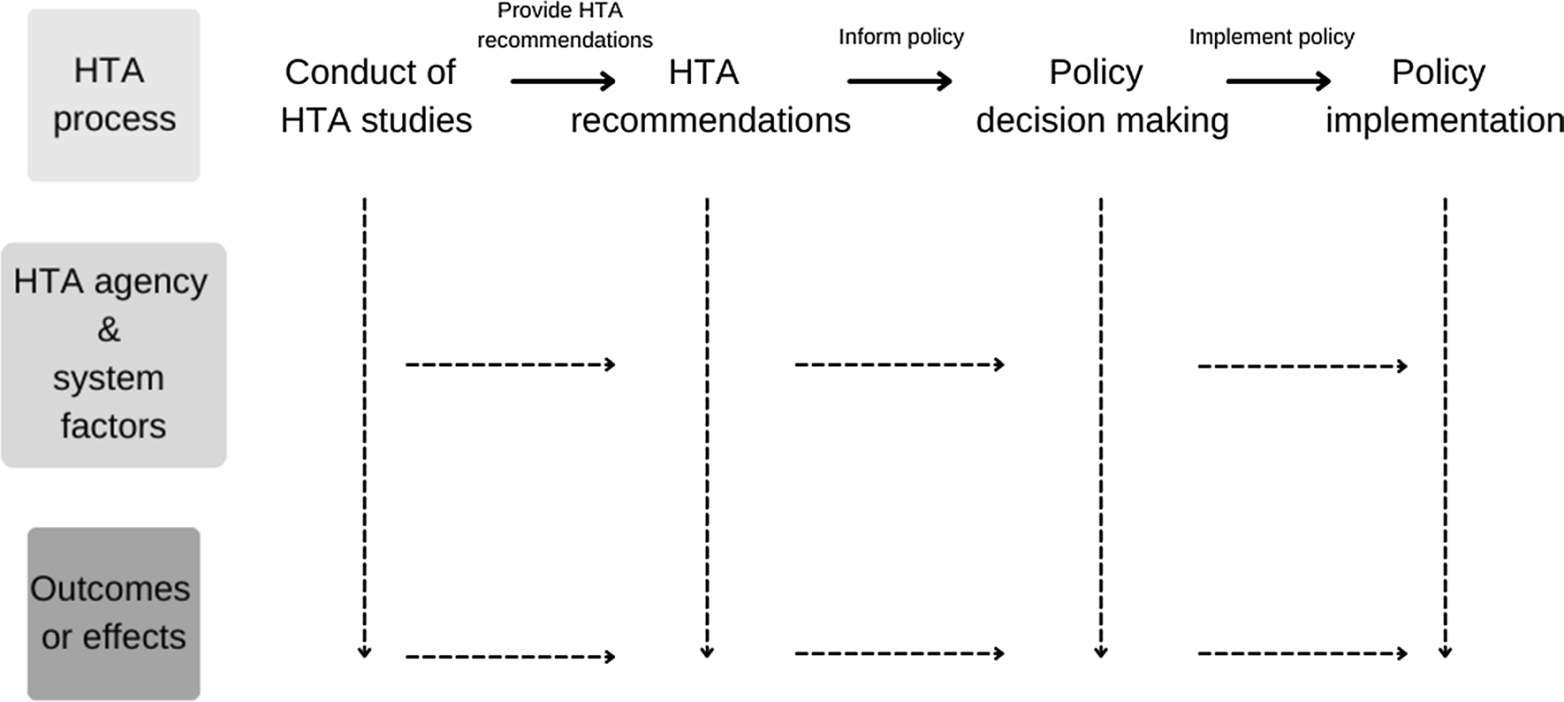
Illustrative example of a logic model structure

We used the interview data to construct logic models for each interviewee. We utilised the logic model format to visualise how each interviewee perceived the specific effects of HTA agencies as well as how each of those effects are realised. This approach resulted in 9 individual logic models, an illustrative example of the structure of which is shown in Fig. 1. The logic model depicts a ‘generic’ process within an HTA system whereby HTA studies are conducted from which HTA agencies make HTA recommendations aimed at influencing policy decision-making and subsequently policy changes are implemented. To account for the multi-dimensional and multi-stakeholder perspective, mechanisms and effects are illustrated throughout and at the bottom of the logic model, respectively.

### Impact mapping

**Fig. 2:**
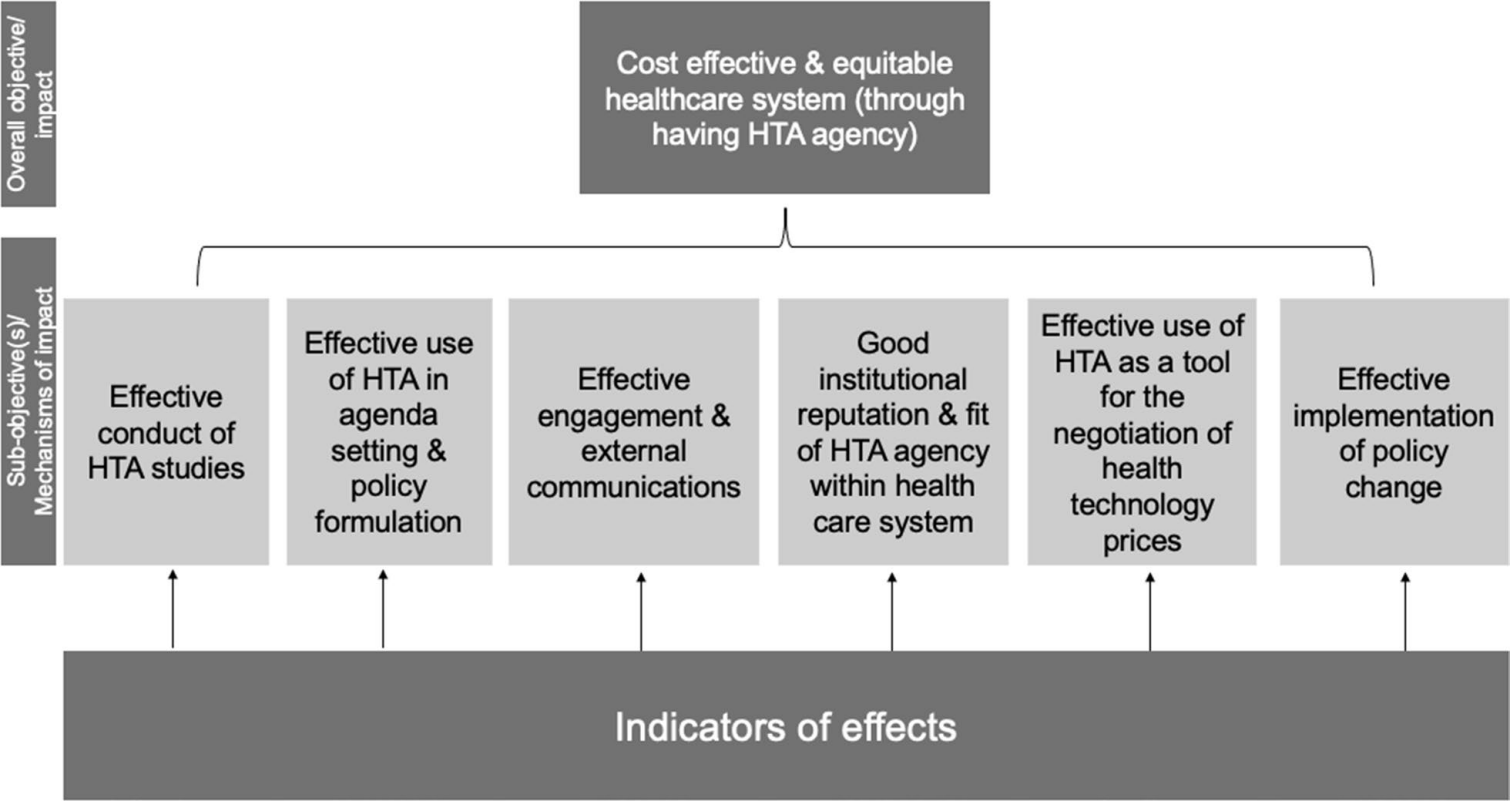
Value tree reflecting impact mapping exercise structure

Using the logic model visualisations of pathways to impact of HTA agencies facilitated a further phase of analysis where we conducted an impact mapping exercise. Specifically, we collated common effects mapped within the individual logic models and, using a backward mapping approach to identify *how* individual effects were perceived to contribute to the overall success of the HTA agency. The reason for doing so was that whilst interviewees were giving their individual perspectives on impact and how impact occurs, it was noted that there was much synergy in the effects realised by the HTA agencies and systems within which they worked despite the fact that different HTA systems were represented in the study. We reflect the learning from this exercise in a value tree, the structure of which is illustrated in Fig. 2. A value tree is a hierarchical map depicting an overall objective with a subsequent layer of sub-objectives (mechanisms of impact) and attributes (which we refer to in this study as ‘indicators of effects’) of those mechanisms of change for a given situation. We discuss the learning from this two-stage analysis in the following results section.

## Results

The value tree in Fig. 2 depicts the overall objective of having an HTA agency, that is to achieve a more cost-effective and equitable healthcare system, at the top of the diagram. The subsequent ‘layer’ of the graph, which we refer to as ‘sub-objectives’, describes several distinct but not necessarily mutually exclusive mechanisms through which the overall objective of an HTA agency is achieved. Following that are specific ‘attributes’ of each sub-objective which reflect in this case indicators of those effects leading to overall impact of an HTA agency from a multi-dimensional and multi-stakeholder perspective: we refer to these attributes in Fig. 2 as ‘indicators of effects’.

More specifically, this graph serves to emphasise that in order for an HTA agency to meet its overall objective of contributing to more cost-effective and equitable health care, there are several ‘sub-objectives’, or mechanisms, through which that overall objective is achieved. These are: the effective conduct of HTA studies; effective use of HTA in agenda-setting and policy formulation processes; effective engagement and external communications; good institutional reputation and fit within the healthcare and policy-making system; effective use of HTA as a tool for the negotiation of health technology prices; and the effective implementation of policy change regarding health technologies. In our mapping, the subsequent ‘layers’ of the graph reflect examples of distinct aspects of each sub-objective which can act as indicators of of whether they have been achieved. Through this we can reflect on both the multi-dimensional and multi-stakeholder nature of the impact of an HTA agency.

**Fig. 3:**
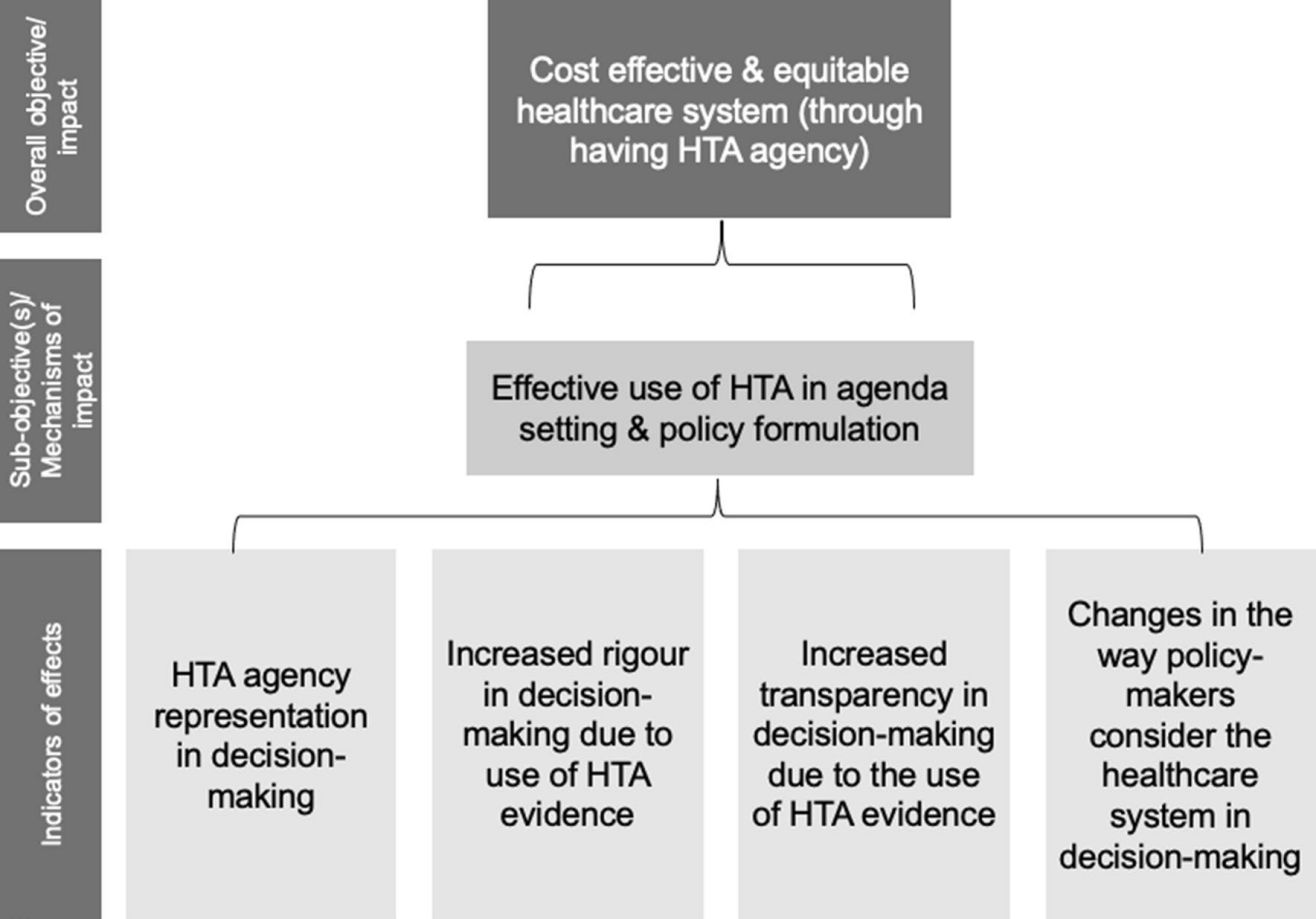
Indicative example of value tree

To take an example, we can look more closely at the sub-objective, or mechanism, of: ‘effective use of HTA in agenda-setting and policy formulation processes’ (shown in Fig. 3). The idea captured here is that through having an HTA agency, HTA can be more effectively used in agenda-setting and policy formulation processes. But how would we know that HTA is being used more effectively as a result of the HTA agency rather than for example because there is some stakeholder with a keen interest in using HTA? Indicators that HTA is being used more effectively in policy making processes as a result of the HTA agency might include the representation of the HTA agency in policy decision-making processes. Moreover, due to the increased use of evidence from HTA studies, the effective use of HTA in policy making might also result in increased rigour in decision-making as well as improved transparency in how policy-makers and insurers decide which health technologies to fund. Also, the more effective use of HTA in policy decision-making through an HTA agency might impact upon the perceptions of policy makers on the healthcare system and the importance of the role of HTA. However, it is worth noting that since our results have been populated with the empirical data collected for this study only, it is thus simply illustrative of the vast array of individual effects which can occur as a result of the presence of an HTA agency across different healthcare systems.

The results of this impact mapping exercise point to lessons and questions which can inform the development of a framework for the evaluation of impact of HTA agencies. Overall, we have learnt it is important that evaluations of the impact of an HTA agency acknowledge the multiple mechanisms through which impact can occur. The role an HTA agency plays in activating each of these mechanisms can have several distinct effects. First, this reflects the potential outcomes HTA agencies can have for specific stakeholders and institutions within the wider healthcare system. The impact mapping exercise therefore highlights that there are multiple and potentially competing effects between stakeholders and institutions in the wider healthcare system that should be adequately acknowledged in evaluations of impact. Second, the impact mapping exercise highlights that the individual effects of a given mechanism, can in many cases be used as indicators, or at least point to questions an evaluation might address with respect to the overall impact of an HTA agency. For example, in understanding how the HTA agency has achieved effective engagement and external communications, an evaluation could address how the work of an HTA agency has challenged social perceptions, and increased awareness and understanding of the challenges facing the healthcare system and the role of HTA in healthcare decision-making through informing public debate in the media.

To complement these findings, we have developed a framework for the evaluation of the impact of an HTA agency which outlines several questions related to the mechanisms of impact derived from the impact mapping exercise and resulting indicators. This framework is shown in Table 1. The mechanisms of impact displayed in Table 1 map directly to those illustrated in the value tree in Fig. 2. In developing the framework we have provided a list of questions related to each mechanism which the reader can use as a template to guide the conduct of HTA agency impact evaluations.

**Table 1:** Framework for evaluating the impact of HTA systems

Mechanism 1: The effective conduct of HTA studies		
Q1	Is the HTA agency doing/commissioning HTA studies that are fit for purpose?	
	Sub-questions: Are HTA studies quality-controlled, for example through submitting them for peer-reviewed publication in international journals, or by having them quality assessed and scored by experts (e.g. in-country academics; colleagues from sister agencyes in other countries)? Does the agency have published technical methods guidance, in order to facilitate quality control by providing a normative standard, and improve methods transparency? Are studies assessed against an international checklist, such as the CHEERS checklist? Do staff have appropriate formal qualifications, and are the duration and resourcing of studies in line with international benchmarks?	[15–17, 29, 33, 35]
	Examples of effects: New method development; availability of good quality and relevant HTA evidence; transparent use of data in research; identification of research gaps; investment in and allocation of resources to HTA research	
Mechanism 2: The effective use of HTA in agenda-setting and policy formulation processes		
Q2	Are HTA studies used in agenda-setting/policy formulation?	
	Sub-questions: Do statute or administrative regulations clearly define who will use the outputs of the HTA agency and how (i.e. are there regulations in place for how HTA studies are to be implemented?), and is it possible to map the process by which HTA studies will impact clinical practice? The usage may be chained, in the sense that, for example, one body might use HTA studies to develop clinical guidelines, and then a further quality assurance body may monitor compliance with clinical guidelines. Is it possible to track study recommendations through the various steps on this process? Are some HTA studies cited outside of this process, for example in policy documents from the Ministry of Health or strategic plans of insurers?	[9–11, 17, 19, 35]
	Examples of effects: HTA agency representation in policy decision-making; increased rigour in policy decision-making due to the use of HTA evidence; transparent policy decision-making due to use of HTA evidence; changes to the way policy decision-makers consider the healthcare system in decision-making	
Mechanism 3: Effective engagement and external communications Mechanism 4: Good institutional reputation and fit within healthcare system		
Q3	Are HTA studies valued by stakeholders in the healthcare system?	
	Sub-questions: Are the various stakeholders in the healthcare system (for example, clinicians, including opinion leaders, manufacturers, managers of insurers and providers, patient groups, academics) aware of studies and do they perceive them to be credible? Where appropriate, do these stakeholders change their behaviour (for example clinical practice, regulatory actions, sales or purchasing) as a result of HTA studies? Is HTA perceived to have influenced conversations between different actors to focus more on evidence of effectiveness and cost-effectiveness? What are the formal mechanisms for engaging these stakeholders in the work of the HTA agency?	[8–11, 15, 17, 19, 35]
	Examples of effects: Use of HTA principles by other stakeholders or by other institutions; acknowledgement of HTA agency recommendations in policy-making processes; increased transparency due to autonomy of HTA agency	
Q4	Do HTA studies inform public debate?	
	Sub-questions: Are HTA studies picked up in non-specialist outlets, such as the public media? Do such studies stimulate public debate (e.g. editorials in mainstream newspapers, interviews or panel discussions in news analysis programmes, documentaries, public events)? Are the public aware of the role of the HTA agency and do they perceive it positively? What mechanisms does the HTA agency have for engaging members of the public in its deliberations?	[8, 33, 35]
	Examples of effects: Increased public understanding of the challenges facing the healthcare system; increased public understanding of the value of HTA and its use in healthcare decision-making	
Mechanism 5: Effective use of HTA as a tool for the negotiation of health technology prices		
Q5	Do HTA studies support insurers or government in negotiation with manufacturers?	
	Sub-questions: Are HTA studies used in negotiations with manufacturers, and do the negotiators report price reductions, or other benefits such as risk-sharing agreements as a result? Do manufacturers litigate HTA agency recommendations (indicating that these recommendations are perceived to drive commercial consequences)?	[10, 12, 17, 33]
	Examples of effects: Number of health technologies funded by public sector after successful price negotiation; healthcare payers can afford to pay for and provide access to health technologies; sustainability of manufacturer income; sustained innovation processes; increased transparency in price negotiation	
Mechanism 6: Effective implementation of policy change		
Q6	Do HTA studies result in changes in practice, and did such changes lead to measurable improvements in cost, health impacts, and wider social/economic impacts?	
	Sub-questions: Is it possible to track activity and prescribing rates (in a consistent way across the country and between subnational entities)? Do recommendations from HTA studies result in observable changes in practice? Is it possible to attribute the change in practice to the HTA agency recommendation (for example a similar change is not observed in other countries where no recommendation has been promulgated)? Is it possible to test key modelling assumptions about costs and health benefits of the technology once the recommendation has been implemented, and verify that the costs and health benefits are in line with what was modelled in the HTA study?	(10–13, 16, 19, 28, 41)
	Examples of effects: Effective uptake of new health technology in practice through effective communication about policy change ensuring acceptability and receptiveness to policy change; improved provision of cost-effective care (including rejection or disinvestment of cost-ineffective health technologies); improved population health; wider social/economic impacts	

The right-hand column provides some examples of studies that addressed or particularly stressed the relevance of the aspects listed in the left-hand side of the table.

## Discussion

Whilst there are a number of studies relating to the question of “what is the impact of health technology assessment?”, there is substantial heterogeneity in this literature with respect to purpose of the study, the conceptualisation of HTA, the conceptualisation of impact, and the scope and rigour of studies. Moreover, studies are often presented from a national rather than international perspective with considerable variations in reporting, making comparisons across countries and contexts difficult. We argue that evaluations could be greatly improved with a few minimally accepted indicators of impact measurement using a systems-focussed framework underpinned by an internationally informed, multi-dimensional, and multi-stakeholder framework. We argue that whilst maintaining the balance between rigour and scope of an evaluation study is incredibly difficult, if not impossible, to conduct, that is not to say that the frameworks guiding such evaluative activity cannot be improved. Realistic approaches to generating knowledge must be taken, for example through addressing a list of questions that are answerable and sufficient to assess the impact of a given HTA agency.

It is worth noting at this point that other studies have developed similar frameworks and our findings are consistent with these frameworks. Based on a systematic programme of qualitative research, the results of this study present a further piece of evidence on how to evaluate the performance of HTA agencies from an institutional perspective rather than viewing HTA as only a ‘knowledge product’. For example, the literature review and interviews published by Charles River Associates [5] compares the use of HTA in several different countries and acknowledges the importance of a multi-stakeholder perspective as well as the lack of evidence on policy and practice in the literature due to a lack of best practice principles for the evaluation of HTA agencies. Similarly, the Payback Framework, the most commonly used model for the evaluation of HTA [4], has a multi-stakeholder perspective but primarily focuses on health services ‘research’. The importance of a multi-stakeholder perspective is also highlighted in national reports from the Austrian and Dutch contexts [14, 22]. However, the aim of this study was to explore the mechanisms of impact of an HTA agency from a multi-dimensional, multi-stakeholder and international perspective with a view to considering the impact of HTA as part of a wider ecosystem of stakeholders, processes, and institutions. With this study, we aim to contribute towards developing a more appropriate framework for the evaluation of HTA agencies.

Learning from our qualitative study contributes to the development of a framework for the evaluation of the impact of HTA agency from an institutional perspective. First, we argue that there are multiple and not necessarily mutually exclusive mechanisms through which HTA systems can meet their overall objective of achieving a more cost-effective and equitable health care system. The HTA agency therefore has a central role to play in ensuring that each of these mechanisms are employed in their work. Moreover, we learn that each of these mechanisms can have distinct features which can act as indicators of effects, or point to important questions evaluations should address. These distinct effects are realised by multiple stakeholders situated within the eco-system of stakeholders, institutions, and processes we have outlined in the introduction. Such learning complements and extends extant literature addressing the question of ‘what is the impact of HTA’ by exploring the mechanisms of impact of an HTA agency from an institutional perspective, rather than viewing HTA as a ‘knowledge product’.

In making this learning practicable for those conducting evaluation activities on the impact of HTA agencies, we have developed framework of questions which evaluators may wish to use to guide evaluation activities, either for a one-off stocktake of the performance of an HTA system, for routine performance monitoring over time, or for comparative benchmarking against other countries. It will be important going forward that this framework of questions is beta-tested in multiple contexts to further refine and ensure the usability of the framework.

### Study limitations

This study has several limitations of note. The substantive component of this study has primarily considered only the healthcare system context, when the impact of an HTA agency will most certainly extent beyond the healthcare system, and into the social care and education systems, for example. Moreover, whilst we adopt an international perspective, we must acknowledge that the majority of interviewees in this sample came from countries which can be classed as developed economies. Further analyses would benefit from a wider range of contextual perspectives. Nevertheless, many of those whom were interviewed had significant international expertise in working with HTA agencies. We must also acknowledge that our empirical work is informed by only 9 senior figures in the international HTA community, meaning that experiences are limited to those interviewed.

## Conclusions

The development of HTA agencies has grown as a result of the increasing importance of HTA research and an acknowledgement of the role of HTA can play in delivering UHC. Understanding the added value of HTA agencies is therefore important when such agencies often divert money from frontline services. This paper offers a complementary perspective to other studies of the evaluation of HTA. In doing so, we adopt an international, multi-stakeholder, and multi-dimensional perspective to explore the mechanisms of impact from an institutional perspective.

The findings of our qualitative study point to several distinct but not necessarily mutually exclusive mechanisms through which HTA agencies can have value, or impact. Our findings inform the development of a framework consisting of several categories of questions which HTA agency stakeholders might wish to address in evaluating the value of their respective agencies, or in considering the development of an HTA agency. Overall, what is clear is that there are multiple mechanisms through which HTA agencies have impact, mechanisms which relate to a wider number of effects for a variety of stakeholders. If the value of HTA agencies are to be realised, then we hope that the framework developed here will serve to support the development of a few minimally accepted indicators of HTA agency impact.

Our conclusions echo and to those from researchers studying the distinct but related question of how to assess the value of HTA *research* programmes. Whereas much of the extant literature perceives the question of ‘what is the impact of HTA’ from the perspective of HTA as a ‘knowledge product’, our study adopts a perspective which conceptualises HTA impact from an institutional perspective, allowing us to explore and identify the specific mechanisms of impact from an HTA agency perspective.

Through having a more detailed understanding of the mechanisms of impact of an HTA agency from an institutional perspective, we hope that our analysis will be useful both to countries interested in managing the performance of their own HTA agencies and benchmarking performance against their peers but also to those development partners who are increasingly funding health systems strengthening initiatives including HTA agencies. For both parties, in order to measure the impact of HTA agencies we need to be able to understand the mechanisms through which the impact occurs. To support this end, we present a framework for the evaluation of the impact of HTA agencies. The framework suggests, based on our impact mapping, several areas of questions which an evaluation of an HTA agency might wish to consider to achieve a fuller understanding of how the impact of their HTA agency has been realised.

## Supplementary Information

**Additional file 1.** Interview guide.

## Data Availability

Not applicable.
