# Supplementary material for: Assessing the performance of health technology assessment (HTA) agencies: developing a multi-country, multi-stakeholder, and multi-dimensional framework to explore mechanisms of impact
Source: Cost Eff Resour Alloc. 2021 Jul 2;19:37. doi: 10.1186/s12962-021-00290-8 (PMC8252304; doi:10.1186/s12962-021-00290-8)
Supplement: Supplementary file 1 — Additional file 1. Interview guide. [file 12962_2021_290_MOESM1_ESM.docx]

**INTERVIEW PROTOCOL**

**INTRODUCTION**

To facilitate our note-taking, we would like to audio tape our conversations today. Only project researchers will have access to these audio recordings which will be stored in password-protected institutional cloud storage. All information will be handled in a confidential manner and your participation is voluntary. Thank you for your agreeing to participate.

You have been approached to speak to us because you have been identified as someone with expertise and experience of both health technology assessment (HTA) and HTA systems within the country you are based. As you will be aware, health technology assessment has major benefits in terms of highlighting the monetary benefits of evidence-informed policy-decisions. However, less research focusses on capturing benefits/impacts beyond these monetary benefits. Through this project, we seek to understand the pathways through which the broader benefits of having a health technology assessment system are realised. We seek to learn from your experience and expertise to understand this. We will use the information gathered to create a logic model.

In this interview we will discuss your experiences of the impacts of an HTA system on several domains of interest. These are research output, future research & research use, impact on policy-making and decision-making processes, health outcomes, health sector, public awareness, social attitudes, economic benefits Within each domain, we will discuss what (if any) the impacts have been, how the impacts were realised, any influences on the processes to impacts being realised, and further consequences of these impacts.

**INTERVIEWEE:________________________________________**

**ORGANISATION:_______________________________________**

**ROLE:_________________________________________________**

Page Break

**INTERVIEW QUESTIONS**

1. **Impact on knowledge i.e. research output**
2. What has been the impact(s) on knowledge, i.e. research output, as a result of having an HTA agency in country X i.e. what has changed;
3. What are the steps through which/how were these change(s) were realised;
4. What were the facilitators/barriers to the change(s) being realised;
5. And were there any other influences on this process, positive and/or negative, internal and/or external to the HTA system?
6. What have the consequences of these changes been?

Page Break

1. **Impact on future research and research use**

1. What has been the impact(s) on future research and research use as a result of having an HTA agency in country X i.e. what has changed;
2. What are the steps through which/how were these change(s) were realised;
3. What were the facilitators/barriers to the change(s) being realised;
4. And were there any other influences on this process, positive and/or negative, internal and/or external to the HTA system?
5. What have the consequences of these changes been?

Page Break

1. **Impact on policy-making and decision-making processes**

1. What has been the impact(s) on policy-making & decision-making processes as a result of having an HTA agency in country X i.e. what has changed;
2. What are the steps through which/how were these change(s) were realised;
3. What were the facilitators/barriers to the change(s) being realised;
4. And were there any other influences on this process, positive and/or negative, internal and/or external to the HTA system?
5. What have the consequences of these changes been?

Page Break

1. **Impact on health and health sector**

**D.1. Health outcomes**

1. What has been the impact(s) on health outcomes as a result of having an HTA agency in country X i.e. what has changed;
2. What are the steps through which/how were these change(s) were realised;
3. What were the facilitators/barriers to the change(s) being realised;
4. And were there any other influences on this process, positive and/or negative, internal and/or external to the HTA system?
5. What have the consequences of these changes been?

**D.2. Health sector**

1. What has been the impact(s) on the health sector as a result of having an HTA agency in country X i.e. what has changed;
2. What are the steps through which/how were these change(s) were realised;
3. What were the facilitators/barriers to the change(s) being realised;
4. And were there any other influences on this process, positive and/or negative, internal and/or external to the HTA system?
5. What have the consequences of these changes been?

Page Break

1. **Broader benefits (**public awareness/social attitudes/economic benefits)

1. Have there been any other impact(s) as a result of having an HTA agency in country X i.e. what has changed (public awareness/social attitudes/economic benefits);
2. What are the steps through which/how were these change(s) were realised;
3. What were the facilitators/barriers to the change(s) being realised;
4. And were there any other influences on this process, positive and/or negative, internal and/or external to the HTA system?
5. What have the consequences of these changes been?

**END**

Do you have anything else you would like to add at this stage? If not, I will conclude the interview and thank-you very much for your time & sharing your expertise with me.
